# P-868. Evaluating the Clinical Impact of Rapid Diagnostic Testing on Antimicrobial Stewardship Interventions

**DOI:** 10.1093/ofid/ofae631.1059

**Published:** 2025-01-29

**Authors:** Ricky Huynh-Phan, Kevin Le, Todd M Lasco, Andrei Zidaru, Vincent Tam, Kady Phe

**Affiliations:** University of Houston, Spring, Texas; Baylor St. Luke's Medical Center, Houston, Texas; Baylor St. Luke's Medical Center, Houston, Texas; Baylor St. Luke's College of Medicine, Houston, Texas; University of Houston, Spring, Texas; Baylor St. Luke's Medical Center, Houston, Texas

## Abstract

**Background:**

The management of bloodstream infections (BSIs) may be improved with rapid diagnostic identification. The updated Biofire FilmArray blood culture identification (BCID) 2 panel is able to detect extended-spectrum beta-lactamase and additional carbapenemase encoding genes amongst other resistance markers, allowing for earlier antimicrobial stewardship interventions. The purpose of this study was to evaluate the time to optimal antimicrobial therapy.

**Methods:**

This was a single-center retrospective chart review of patients before and after the implementation of the updated BCID panel (BCID1 vs. BCID2, respectively). Adult patients were eligible for inclusion if they had a positive blood culture with an accompanying BCID panel result. Patients were excluded if they received concomitant treatment for an unrelated infection or had monomicrobial BSI with *Staphylococcus* species. The primary endpoint was time to initiation of optimal antimicrobial therapy from index blood culture, defined as pathogen and resistance gene directed therapy per hospital treatment algorithm. Secondary endpoints included time to active antimicrobial therapy, defined as empiric therapy shown to be active against index isolate(s) with an antimicrobial susceptibility report, and 30-day mortality.

**Results:**

Overall, 143 patients were included in the BCID1 group and 175 patients in the BCID2 group. Time to optimal antimicrobial therapy was shorter in the BCID2 group compared to the BCID1 group (17.1 hours vs. 29.3 hours; p < 0.05). Time to active therapy was shorter in the BCID2 group compared to the BCID1 group (3.1 hours vs. 5.9 hours; p=0.03). Thirty-day mortality rate was lower in the BCID2 group compared to the BCID1 group (9.7% vs. 20.9%; p < 0.05).

**Conclusion:**

Time to optimal and active antimicrobial therapy were significantly shorter after the implementation of the BCID2 panel. Furthermore, 30-day mortality was lower, indicating that optimized therapy per local hospital treatment algorithms may improve outcomes.

**Disclosures:**

**All Authors**: No reported disclosures

